# Shear Forces during Blast, Not Abrupt Changes in Pressure Alone, Generate Calcium Activity in Human Brain Cells

**DOI:** 10.1371/journal.pone.0039421

**Published:** 2012-06-29

**Authors:** Rea Ravin, Paul S. Blank, Alex Steinkamp, Shay M. Rappaport, Nitay Ravin, Ludmila Bezrukov, Hugo Guerrero-Cazares, Alfredo Quinones-Hinojosa, Sergey M. Bezrukov, Joshua Zimmerberg

**Affiliations:** 1 Program in Physical Biology, Eunice Kennedy Shriver National Institute of Child Health and Human Development, National Institutes of Health, Bethesda, Maryland, United States of America; 2 Center for Neuroscience and Regenerative Medicine at the Uniformed Services University of the Health Sciences, Bethesda, Maryland, United States of America; 3 Department of Neurosurgery, Johns Hopkins University, Baltimore, Maryland, United States of America; Dalhousie University, Canada

## Abstract

Blast-Induced Traumatic Brain Injury (bTBI) describes a spectrum of injuries caused by an explosive force that results in changes in brain function. The mechanism responsible for primary bTBI following a blast shockwave remains unknown. We have developed a pneumatic device that delivers shockwaves, similar to those known to induce bTBI, within a chamber optimal for fluorescence microscopy. Abrupt changes in pressure can be created with and without the presence of shear forces at the surface of cells. In primary cultures of human central nervous system cells, the cellular calcium response to shockwaves alone was negligible. Even when the applied pressure reached 15 atm, there was no damage or excitation, unless concomitant shear forces, peaking between 0.3 to 0.7 Pa, were present at the cell surface. The probability of cellular injury in response to a shockwave was low and cell survival was unaffected 20 hours after shockwave exposure.

## Introduction

Traumatic Brain Injury (TBI) is a major public health problem. Since 2001, over 150,000 US military personnel have been diagnosed with a mild form of TBI, often after exposure to an explosive blast (bTBI), with a spectrum of neurological and psychological deficits [1], [2], [3]. Mild bTBI is enigmatic, hard to diagnose, often without external injuries, and often goes un- or misdiagnosed [4]. The mechanisms of the primary injury phase, a direct result of the shockwave generated by an explosion, are the least understood [5], [6], [7]. The blast shock wave (BSW) of primary bTBI is a transient, solitary supersonic pressure wave with a rapid (sub-msec) increase in pressure (*i.e.* compression) followed by a more slowly developing (msec) rarefraction phase of low pressure (*i.e.* tension) [8]. In the majority of bTBI, the peak pressure is low; exposure to blasts estimated to create 10 atm peak pressure in the skull for a few milliseconds can result in death for unprotected persons [9]. Although dynamic compression, tension, and shear stress have all been proposed to explain primary bTBI [5], the identity of the mechanical forces involved, the tissue-force interaction(s) and the cellular damage properties remain unresolved. Studies on a mechanical head model demonstrated high transient intracranial pressures; shear stresses were not significant [10]. However, finite element mesh simulations produce high shear stresses [11] together with pressure [12]. Intracranial pressures were measured [13] but not intracranial shear stresses. Animal studies on the effects of shockwave *in vivo* [14], [15], [16], [17] are useful for studying aspects of cellular damage mechanisms, but lack real time monitoring of cellular behavior during and immediately after the blast, and fail to decouple the proposed direct effects of the pressure transient from the secondary effects of the shear stresses produced by that pressure transient. *In vitro* models of primary blast injury [5], [18], [19] are likewise limited by an absence of real-time, high spatial and temporal detection of cellular responses during, immediately after, and long after (survival) the blast; none differentiate shear from pressure [20]. Thus, we asked central question: does the BSW itself act directly on human brain cells, or does the BSW act indirectly through secondary shear stresses?

## Results

**Figure 1:**
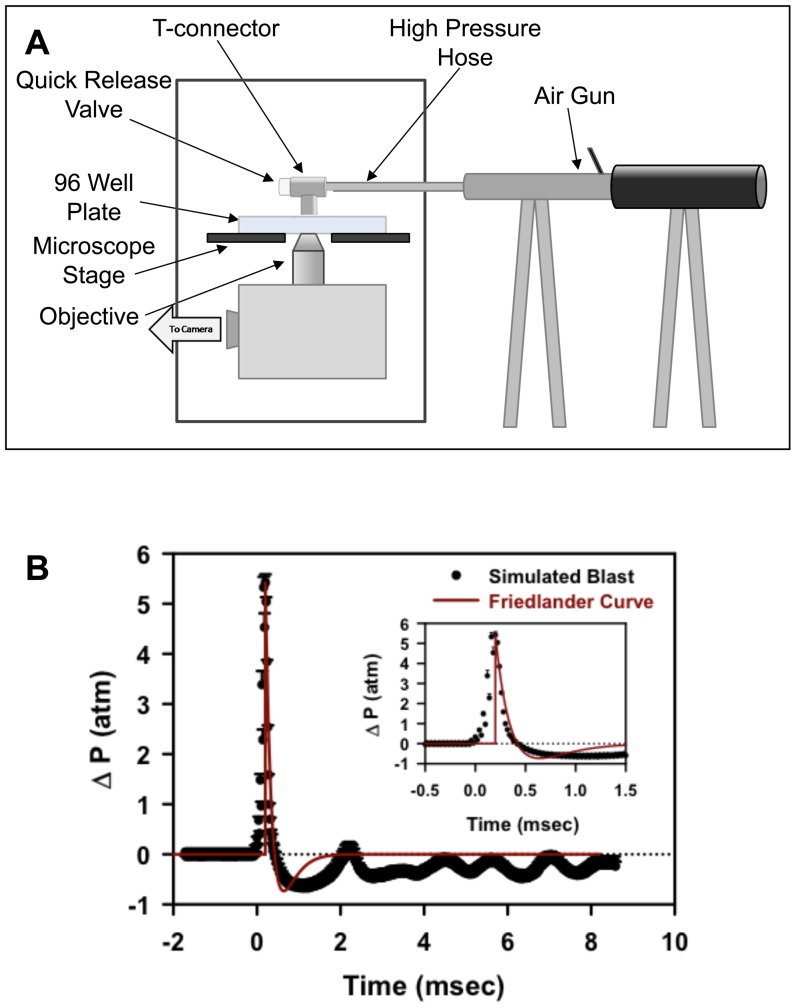
(A) Schematic diagram of the pneumatic device and modified 96 well plate attached to a microscope stage (**B**) Pressure profile measurements of the simulated open field blast shockwave compared to a classical Friedlander curve of the same peak pressure and positive phase duration. Average of 6 measurements is shown with standard error.

We attached a pneumatic device to one of 96 wells positioned on a microscope and vary the amplitude of the pressure transient with an adjustable quick release plug (Figure 1A). The pressure waveform characteristics are comparable to those recorded in open field blasts; the pressure waveform profile closely resembles a classic Friedlander curve [8] (Figure 1B and Text S1). The simulated blasts were generated with rise times in the 0.1 msec range and a two component falling phase: a fast component dropping below ambient pressure within 0.5 msec, and a slower component returning to ambient pressure within 2 msecs (Figure 1B); pressures from 5–14 atm above ambient pressure were examined.

**Figure 2:**
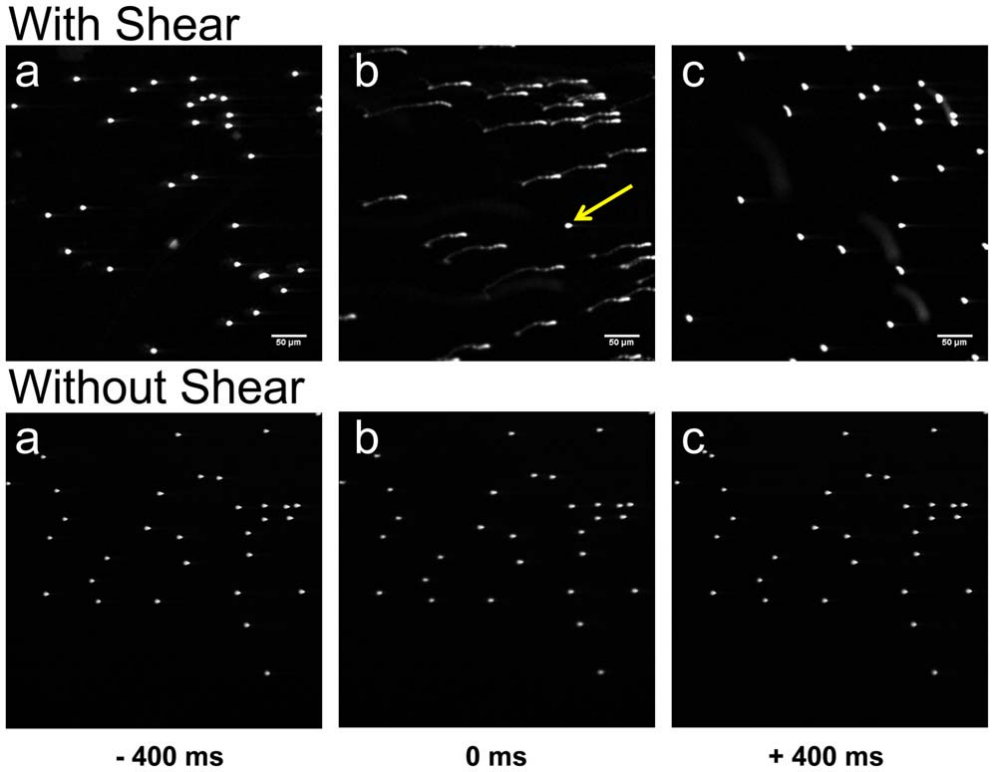
Shear forces are regulated by well fluid volume. Three consecutive frames of 400 msec duration captured beads before (a), during (b), and after (c) the application of a ∼11 atm peak pressure blast with (180 µl fluid volume) and without shear (380 µl fluid volume). Significant bead motion due to shear is registered in top frame b. Note, a single bead did not move (arrow); this bead, presumably immobilized due to adhesion to the surface, allows one to check for stability of the stage during the blast. In the absence of shear, the application of the same peak pressure blast does not show any bead displacement (bottom frame b).

Each blast created not only a fast transient pressure wave, but also shear forces originating at the interface of the gas and liquid. To control the magnitude of shear forces at the cell surface, the well volume was manipulated as larger media volumes increased the distance between cells and the gas-liquid interface, thus reducing the shear forces acting on the cells. To measure the shear forces within the cell plane, fluorescent beads (Molecular Probes L-14822 component E) were used as reference markers on the bottom of wells with varying fluid volumes and subjected to blast (peak pressure 11 atm (10 atm above ambient pressure), Videos S1 and S2). Beads in wells containing 150 and 180 µl exhibited fast motion in response to a blast (Figure 2, With Shear) but beads in 380 µl exhibited no motion with blast (Figure 2, Without Shear). Hereafter, these two volume ranges will be referred to as with and without shear. Analyzing only those trajectories that were two-dimensional and had no self- or inter-crossings, an estimate of the shear stress during a significant part of the impulse was calculated and found to be ∼0.2 Pa averaged over 70 msec with peak shear stress <1 Pa (Text S1 and Figures S3 and S4). In all measurements without shear the velocities and stresses were lower than the lowest shear that could be estimated by our technique, <0.0001 Pa.

**Figure 3:**
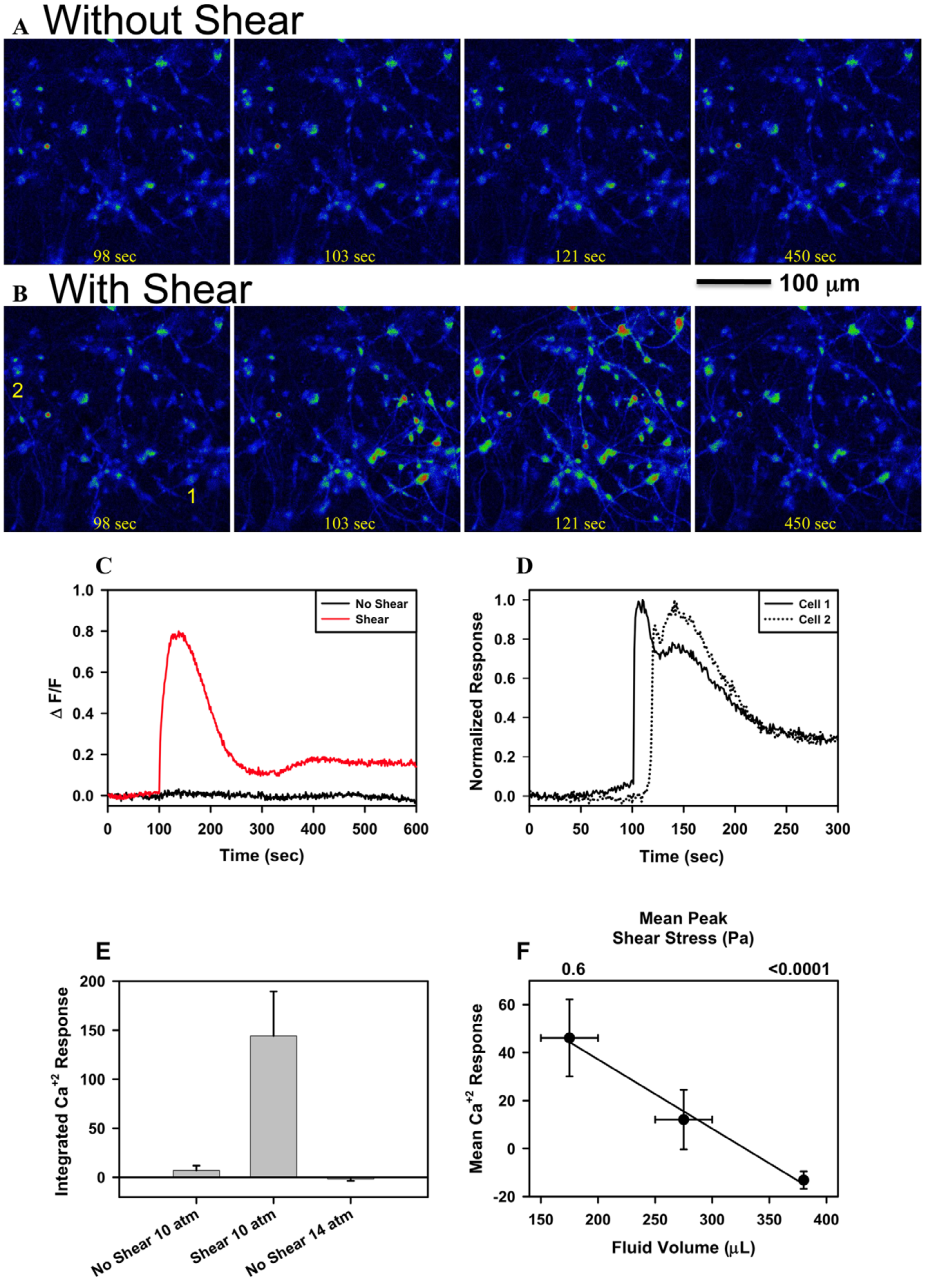
The effect of shear forces on the response of cells to an ∼**11 atm peak pressure simulated blast.** (**A**) Sequential fluo-4 calcium imaging of a dissociated primary human fetal CNS cell culture without shear (380 µl well fluid volume); the blast occurred at time 100 seconds. (**B**) Sequential fluo-4 calcium imaging of the same cells as in Figure 3A with shear (150 µl well fluid volume) using the same blast parameters; the blast occurred at time 100 seconds. (**C**) ΔF/F in time of the cells shown in Figures A and B for 10 minutes before, during, and after the blast. (**D**) Normalized response of two cells shown in Figure B, panel 1, indicating that the calcium response does not occur simultaneously in cells but sequentially; time to peak response is offset by ∼30 seconds in this example and is consistent with propagation (see Video S4). (**E**) Integrated Ca^2+^ Response, integral of ΔF/F over time following the blast, without and with shear forces in the same well, for n = 6 pair-matched experiments (11 atm; 10 atm above ambient pressure). The first blast was without shear forces. The response to a lethal peak pressure of 15 atm (14 atm above ambient pressure) with no shear forces is also shown (n = 10) (**F**) Correlation (r^2^ = 0.99) between Integrated Ca^2+^ Response following a 11 atm peak pressure blast and well fluid volume for the 3 volume conditions evaluated; 150–200, 250–300, and 380 µl with n = 14, 7 and 25, respectively. Fluid volume and Ca^2+^ Response error bars are the range and SEM, respectively.

Cellular calcium signaling is observed only following a blast in the presence of shear. Fields of cells, containing on average 40 cells, were subjected to blast with a peak overpressure of 11 atm first in the absence and then in the presence of shear (Figures 3A and 3B; Videos S3 and S4). Increases in calcium signaling, evaluated using Fluo-4 fluorescence intensity was only observed with shear (Figure 3C). This increase in calcium signaling does not occur simultaneously in all cells but propagates through the system (Figure 3D and Video S4). The average calcium response of cells first exposed to a blast without shear forces and then a blast with shear forces was significantly different (p<0.01, n = 6 paired; Figure 3E). Even when peak pressure exceeded 15 atm (14 atm above ambient pressure, n = 10), a level that is typically lethal to blast victims, cellular signaling was not observed (Figure 3E). The mean calcium response increased with shear (Figure 3F, n = 14, 7, 25).

The role of cellular injury was tested by subjecting cells to a peak overpressure of 11 atm with (n = 12) and without (n = 12) shear in the presence of 100 µM calcein; overpressure conditions in which a calcium response was observed with shear. After controlling for pre-labeling (see Materials and Methods), the appearance of labeled cells following a blast was evaluated in an ∼6.25 mm^2^ area containing ∼2,000 cells. Two types of injured cells were observed; 1) in 2/12 experiments with shear calcein-permeabilized cells along the edge of a cell lifted region and having a labeling pattern analogous to our positive control, scratch wounding, and 2) in all experiments, with (12/12) and without (12/12) shear, a low number (0–6 cells total in 49 fields) of individual calcein-permeabilized cells in an otherwise unperturbed area of the image field. The average number of individual cells wounded in the total area was 2.33 (with shear) and 1.50 (without shear) suggesting a low probability event that can be described by Poisson statistics; the observed frequency distributions are not significantly different from the Poisson distribution with parameters 2.33 and 1.50 (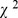, p = 0.05; parameter 95% confidence +/−0.86 and +/−0.69 respectively); the average number of individual, calcein-permeabilized cells detected with and without shear is not statistically different.

Conventional MRI images of victims suffering from mild bTBI show no regions of necrosis or edema [21] suggesting that acute cell death is not the cause of the observed bTBI symptoms. We analyzed the correlation between calcium load and cell survival for a period up to 20 hours following blast exposure with survival evaluated 20 hours after the blast (see Materials and Methods). There is no correlation between cell survival and blasts with or without shear forces; the regression slope is not significantly different from zero, p<0.01 (Figure S2), nor was survival altered in two mock experiments (380 µl volume but no blast) which were not statistically different from the mean survival of all blasts, p<0.01. The mean survival at 20 hours, evaluating 9,120 cells, was 94.7% +/−2.6% and ranged from 91.7%–99.2% (n = 11 experiments).

## Discussion

Our findings show that human brain cells in culture are indifferent to blast induced fast transient pressure waves (BSW) consisting of sub-msec rise time, positive peaks of up to 15 atm, followed by tensions of 0.2 atm, of msec total duration. Furthermore, we have shown that the cells only respond with global elevations in intracellular free Ca^2+^ when sufficient shear forces are simultaneously induced with the pressure profiles. These results makes it unlikely that the primary effect of a BSW on brain cells *in vivo* is a direct effect of the compression and tension forces created by the pressure transient per se. While the pressure transient that is created in our system is very similar to the classic Friedlander curve, it is possible that significant differences exist between the nature of the shear forces created by our system and those induced during an actual blast *in vivo*. In addition, we do not know the magnitudes of the low-pressure components that develop at brain cells during an actual blast. However, the observed correlations between cellular response and shear forces, and the lack of correlation to pressure, *per se*, suggest that shear forces are likely involved in the primary injury phase of bTBI.

The human brain, in its bony skull, is a complex system with multiple inhomogeneities, through which pressure waves travel at different speeds. It is this difference in speed that creates shear, potentially between brain cells [19]. Depending on the orientation of their CNS tissues with the blast propagation, different shear forces may develop and the shockwave may encounter membrane interfaces with different susceptibility to damage. Thus, this is in agreement with observations both in *in vivo* models, as well as with human injuries in which expression of bTBI symptoms among different individuals that are exposed to the same blast is heterogeneous.

The influence of a controlled shear stress on cells in general and neurons in particular has been investigated in a variety of model systems including a rotating cone [22], [23], linear actuator [24], and micro-fluidic-vacuum transfection [25]. However, it is unknown if the kinetics of shear stress produced in these model systems is comparable to that produced in brain as a result of a blast shock wave. Cells survive shear stresses up to 14 *Pa* with 20 msec and longer rise times but permeability of their membranes to soluble dyes and electrical activity is altered in neuronal cultures [22]. Homogenization of brain tissue begins with a power driven pestle creating macroscopic shearing that we estimate at ∼10 *Pa*. Yet such shear forces are insufficient to detach the pre-synaptic terminal from the post-synaptic terminal, thus the adhesion of the interneuronal junction surpasses that of cellular membrane integrity, and the tubular necks of membrane that attach the synapse to the axon, dendrite or cell body preferentially rupture instead, yielding the synaptosome [26]. These properties of the synaptosome preparation suggest that shear stress can act directly at non-synaptic cell membrane. The appearance of a very small number of calcein-postitive cells following a peak overpressure of 11 atm with and without shear suggests that these cells are not the originators of the observed propagated calcium response. However, identification of the originators of the calcium response may be below our detection limit if these cells respond with localized, non-lethal, transient permeabilization and/or activation of shear dependent channel activity. Alternatively, the injured cells responsible for the calcium response were still outside the examined field of view.

Calcium has been implicated in the induction of neuronal death during TBI and stroke; calcium is elevated for long periods of time (days, in cells surviving TBI and stroke [27]). In our experiments, calcium is elevated transiently for short periods of time (seconds to a few minutes), and cell death does not occur even after 20 hours following this excitation. Thus the mechanism of mild bTBI may differ from that in TBI and stroke injuries, which do lead to cell death. Brain cells exposed to blast wave profiles lacking shear forces had no calcium response, even at peak pressures up to 15 atm and trough pressures of 0.2 atm, suggesting that a shear dependent mechanism of primary bTBI may involve mechano-sensitive channels, lipidic pores, or uniquely vulnerable regions of the neuronal plasma membrane, leading to activation of a small population of cells and subsequent amplification through cell-cell signaling. The high curvature stress at the necks of pre-synaptic and post-synaptic boutons or fine processes of astrocytes may be an example of vulnerable regions since the curvature stress would add to the shear stress at those points, known to disassemble during homogenization.

Using primary human brain cell cultures at the level of single and small networks of cells, we found that shear forces acting at cellular length scales, rather than changes in pressure *per se* control the major activation parameter of CNS derived cell culture, intracellular calcium. Rapid compression and positive tension alone have been ruled out as the origin of calcium dependent cell-cell signaling following a BSW. It is now possible to evaluate both the pharmacology of the propagated calcium response associated with a blast in the presence of shear forces and the behavior of other cellular markers during varied blast conditions.

## Materials and Methods

### Ethics Statement

Primary, human central nervous system (CNS) tissue, gestational weeks 19–21, were obtained under surgical written consent according to National Institutes of Health Institutional Review Board Exempt # 5116 under Johns Hopkins University approved protocols, based on its designation as pathological waste.

### Cell Culture

The tops of wells in optical coverslip-bottomed 96 well plates (Granier) were threaded using a 1/16 NPT tap. The 96 well plates were coated with PureCol (Advanced BioMatrix, San Diego, CA; 6 µg/ml). The PureCol coated plates were then coated with poly-D-lysine (Sigma-Aldrich; 10 µg/ml). Primary, human central nervous system (CNS) tissue was dissociated using gentle titration in HBSS, centrifuged, washed and resuspended in neurobasal medium (NB) supplemented with B27 (Invitrogen). Cells were plated at a density of 50,000/well in NB + B27; half of the media volume was changed twice a week. Cells cultured for 2 to 5 weeks were used in all experiments. Cell preparations from seven different tissue specimens were used during this study. Each experiment involved an individual well in a plate. A total of 96 individual wells from these seven plates were used and the number of repetitions for each experiment is indicated in the text. Depending upon the experimental design, the tiled field of view for a single experiment contained ∼2000 cells (7×7 tiling) while the untiled time series observations contained, on average, 40 cells.

### Imaging System

The real time imaging system is a Ti wide-field inverted microscope with Perfect Focus (Nikon, Inc.) equipped with an EM camera (Andor Technology DU-897E) running NIS Elements (Nikon, Inc.) for data acquisition and experimental control. A 20× air objective (NA 0.75) was used for imaging. The fluorescence indicator, Fluo-4 (Invitrogen) was used to monitor intracellular calcium. Fluo-4 was excited using 480 nm light (Chroma HQ 480/40 nm filter) and fluorescence emission collected (Chroma HQ 535/50 nm filter) during an exposure time of 80–100 msec. The image acquisition rate was 1 Hz. An external isolation box (Oko Lab) maintained temperature (37 C) and blocked ambient light. A 96 well plate was mounted on the microscope stage using a custom made clamp designed by the authors and fabricated in house. The microscope condenser was removed to allow access for connecting the pneumatic device to the 96 well plates.

### Calcium Imaging

Before each experiment, cells were loaded in the incubator with 6 µM Fluo-4 AM in NB+B27 and maintained at 37 C with 95% air and 5% CO_2_ for 30 minutes. At the end of the loading period, the cells were washed twice with NB+B27. An additional wash to final well volume was done prior to imaging each well. Prior to initiating blast experiments, the covers of the 96 well plates were replaced by a gas tight film (TSS-RTQ-100 EXCEL Scientific) sealing the wells of the plate, preserving the gas environment inside each well. The 96 well plate cover was replaced with film in a hood and elevated using a plastic spacer attached to a wire. When placed back in the incubator and equilibrated, the wire was used to remove the spacer allowing the film to fall and seal the 96 well plate. Upon removal from the incubator, the film was tightly secured prior to mounting in the microscope. The 96 well plate was attached securely to the motorized stage of the microscope. For each experiment, a single well was centered in the microscope’s field of view; the piece of film covering that well was cut and removed without affecting the sealing of the other wells; a third wash established final well volume. The T-connector was then screwed into the well using the previously tapped threads. The high-pressure tubing was attached to the T-connector using a quick release connection. A quick release valve filter was secured in the T-connector with the other end of the high-pressure hose attached to the pneumatic device. Baseline images were collected every second for 100 seconds. The blast was triggered after the 100^th^ image while continuously imaging the well for 10 minutes. Calcium signaling was evaluated over the entire field of view by averaging fluorescence, ΔF/F as a function of time and then integrating the area under the ΔF/F curve for each experiment.

### Pneumatic Device

The pneumatic device (Figure 1A) is based on an air gun (Airforce Model R9901) that was modified by Axiom/Lemak (Virginia) according to our specifications. This model air gun has a pressurized tank that can be filled with any gas mixture up to a pressure of 4,500 psi. A defined volume of gas is released through an impact valve that is activated by a trigger mechanism. The properties of the gas released (speed, volume, and timing) can be precisely and reproducibly controlled. Modifications included replacing the gun barrel with an 8-inch tube having a high-pressure hose quick connection, modifying the hammer and the spring mechanism that controls the hammer trigger, and changing the gas tank valve. The gas mixture in the pneumatic device, which was pressurized to 1500 psi, was 95% air and 5% CO_2_. To simulate an open field blast the middle male connector threaded vertically into the well while the remaining two female connectors ran horizontally above the well (Figures 1A and S1). One female connector was attached to the high-pressure hose while the other connector was used for the release valve. To best simulate an explosive blast, it was necessary to develop a quick release valve. We designed a simple and reproducible quick release valve using a filter plug taken from a 1250 µl pipette tip (Thermo) that was inserted into the threads of the remaining female connector (Figures 1A and S1) using 1/6–1 ¼ turns resulted in 6–15 atm peak overpressure above ambient pressure. The quick release valve is required in this design because a fixed pressure release valve had a significantly longer rise time. When the air gun trigger is pulled, the device is activated, releasing a burst of pressurized gas from the tank. As the pressurized gas is released, the quick release valve on the T-connector traps gas and a rapid increase in pressure is created within the well. When the pressure exceeds the limit of the quick release valve the plug is ejected, allowing the gas to escape, and resulting in a rapid decay of overpressure as the accumulated pressure is released. The rapid gas flow through the narrowing horizontal portion of the T-connector creates a drop in pressure in the well (Venturi effect), to below ambient levels.

### Injury Analysis

In order to evaluate cell injury following a blast, calcein (Invitrogen) uptake was measured. Cells were plated as described, incubated with 100 µM calcein for 5 min., and washed 4 times. Calcein was imaged using the Fluo-4 settings. An area of ∼6.25 mm^2^ was imaged by tiling 49 regions (7×7 grid containing ∼2000 cells) in order to establish the pre-blast level of calcein labeling. 100 µM calcein was added back to the well and the cells subjected to blast with or without shear and incubated for another 5 min. The cells were washed 4× on the microscope and the same area reimaged. The paired images were background subtracted, aligned, color coded for pre and post blast, and the appearance of new calcein labeled cells was determined. The positive control, for labeling, was scratch wounding the cell layer; cells and cell processes along the scratch were labeled (n = 5). An additional injury control was the same peak overpressure (11 atm) without shear (n = 12).

### Survival Analysis

In order to evaluate cell survival following a blast, a dual color staining of nuclei was used to differentiate between cells that were dead before the blast and those cells that died during the 20 hours after the blast. Cells were loaded with 6 µM Fluo-4 AM for 30 minutes, washed with NB + B27 containing 0.5 µM ethidium homodimer-1 (Invitrogen) in order to stain existing dead cells, incubated for 5 minutes and then washed 3 times with NB + B27. Wells were set with different volumes of NB + B27 in order to establish conditions for a blast with (150 and 180 µl) and without shear (380 µl). Following the blast protocol, the 96 well plate was maintained on the microscope in a custom designed chamber (Precision Plastics, MD) that was maintained at 37 C and 95% air 5% CO_2_. 20 hours after a blast the cells were labeled with 6 µM Fluo-4 AM for 30 minutes, and then 0.5 µg/ml DAPI for 5 minutes in order to identify newly dead cells, washed 2× with NB+B27, and every area previously imaged was reimaged. A total of 25 areas were tiled for every well. The conditions for Fluo-4 imaging were those described previously. DAPI was excited using 350 nm light (Chroma AT 350/50 nm filter) and fluorescence emission collected (Chroma D460/50 nm filter). Ethidium Homodimer-1 was excited using 545 nm light (Chroma HQ 545/30 nm filter) and fluorescence emission collected (Chroma HQ 620/60 nm filter).

ImageJ was used for all image processing. The ethidium homobromide-1 channel (red) and the DAPI channel (blue) were background subtracted, filtered using 2 pixels Gaussian blurring, and a binary image created using Otsu threshold. The binary images were color-coded using red and blue look up tables and merged into one image. Newly dead cells were identified as having nuclei uniquely blue while all previously ethidium homobromide-1 labeled nuclei appeared magenta (blue + red). The merged images were color threshold and the remaining blue nuclei counted using Analyze Particles with the following settings: size 25– infinity (pixel^2^), circularity 0.85–1.00, and exclude on edges. Images were background subtracted, filtered using 2 pixels Gaussian blurring, and Otsu threshold. Cells were counted using Analyze Particles with the following settings: size 100– infinity (pixel^2^), circularity 0–1.00, and exclude on edges. The total cell count was the sum of newly dead cells (uniquely blue nuclei) and the Fluo-4 cell count. The survival fraction at 20 hours is expressed as the ratio of blue nuclei to the total cell count (Fluo-4 cell count + uniquely blue nuclei).

### Shear Analysis

In order to estimate the shear stress produced by the pressure pulse, 6 µ*m* InSpeck fluorescent (Invitrogen, Inc.) component E microspheres (beads) were added to identical 96 well plates containing phosphate buffered saline (PBS); conditions, such as temperature and relative positions in the chambers were the same as in the cell experiments. During acquisition of a continuous series of frames with 400 *ms* exposure time each, the pressure pulse was applied to wells with different fluid volumes. The trajectory of a moving bead appears in one of the frames as a continuous curve of varying intensity (see Figure 2). The position-dependent brightness of a particular track is inversely proportional to the bead velocity along the track. The exposure time and camera gain were selected guided by three main goals: capturing the bead movement in one frame, detecting sufficient light during the highest bead velocity, and minimizing camera saturation at low velocities. The correlation between light intensity and exposure time is calculated by averaging the intensities of static beads using different exposure times in the range of 10–400 *ms*. This calibration is used to convert intensities of the moving beads into time units and define the arc length (i.e., the distance along the trajectory) of the bead trajectories over time. The velocity is derived using the first derivative of the arc length with respect to time. An approximate linear relation between the flow velocity and separation *z* from the surface is then used to estimate the shear stress (see Text S1). The main assumptions are: (i) the beads move together with the liquid (i.e., Brownian motions are negligible); (ii) the shear stress sensed by a cell is only due to the flow of media that is close to the chamber surface, *z* <6 µ*m*; (iii) the beads used to evaluate flow have a quasi-two dimensional path (i.e., move mostly in the *x−y* plane and not the *z*-direction). In all experiments analyzed there is a clear correlation between trajectories of the moving beads, justifying the assumption that for short time periods after the blast the beads move together with the surrounding liquid. However, the bead trajectory characteristics varied; in some experiments the trajectories were three-dimensional with beads moving out of the focal plane. This behavior may be related to turbulence. Even when most beads moved, a few beads exhibited little or no movement. This behavior may be related to strong surface adhesion. Images were processed with ImageJ (NIH) and analyzed with Origin 8.51.

## Supporting Information

Figure S1 Schematic diagram of the T-connector installed onto the mock well chamber and fiber optic pressure sensor.
(TIF)

Figure S2 Cell survival is independent of blast conditions. A) ΔF/F for three examples of blast conditions, with and without shear and mock, no blast; survival at 20 hours was comparable for all three conditions, greater than ∼94%. B) The mean survival at 20 hours, evaluating 9,120 cells, was 94.7% +/−2.6% and ranged from 91.7%–99.2% with no correlation between survival and shear or blast (n = 11 experiments).
(TIF)

Figure S3Stationary bead intensity as a function of exposure time. Error bars are standard deviations, and the solid line is the best fit to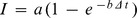 (Eq. (1)) with *a* = (8.73±0.25)×103 and *b* = (4.95±0.29)×10−3.(TIF)

Figure S4 Derivation of shear stress profiles and representative examples. A) Intensity vs. trajectory length (Distance) for a moving bead captured in one image frame (see Figure 2). B) Time dependent trajectory length (Distance) vs. Time calculated using Eq. (2) and Eq. (3). C) Velocity and shear stress derived from the derivative of the time dependent trajectory length (thick red curve) and four other representative examples.
(TIF)

Text S1 Supporting information text for “Shear Forces During Blast, Not Abrupt Changes in Pressure Alone, Generate Calcium Activity in Human Brain Cells” by Rea Ravin, Paul S. Blank, Alex Steinkamp, Shay Rappaport, Nitay Ravin, Ludmila Bezrukov, Hugo Guerrero-Cazares, Alfredo Quinones-Hinojosa, Sergey M. Bezrukov, and Joshua Zimmerberg.
(DOC)

Video S1 Fluorescent beads subjected to a blast shock wave with shear. The fluid volume in the well was 180 µl and the peak overpressure was 11 atm. Under these conditions, movement of the beads can be detected in the movie. The shear forces can be calculated from the motion observed in the individual image frames.
(AVI)

Video S2 Fluorescent beads subjected to a blast shock wave without shear. The fluid volume in the well was 380 µl and the peak overpressure was 11 atm; all the blast parameters were identical to those used in Video S1 except for the well fluid volume. Under this condition, no bead motion was observed and the shear was estimated to be less than 0.0001 Pa.
(AVI)

Video S3 Calcium imaging of cells labeled with Fluo-4 subjected to a blast shock wave without shear. The fluid volume in the well was 380 µl and the peak overpressure was 11 atm. Under this condition, there was no calcium response to the shock wave.
(AVI)

Video S4 Calcium imaging of cells labeled with Fluo-4 subjected to a blast shock wave with shear using the same field of cells recorded in Video S3. The fluid volume was 180 µl and the peak blast overpressure was 11 atm; all the blast parameters were identical to those used in Video S3 except for the well fluid volume. Under this condition, there was a calcium response to a blast shock wave.
(AVI)
