# Supplementary figures and images for: Shear Forces during Blast, Not Abrupt Changes in Pressure Alone, Generate Calcium Activity in Human Brain Cells
Source: PLoS One. 2012 Jun 29;7(6):e39421. doi: 10.1371/journal.pone.0039421 (PMC3387147; doi:10.1371/journal.pone.0039421)

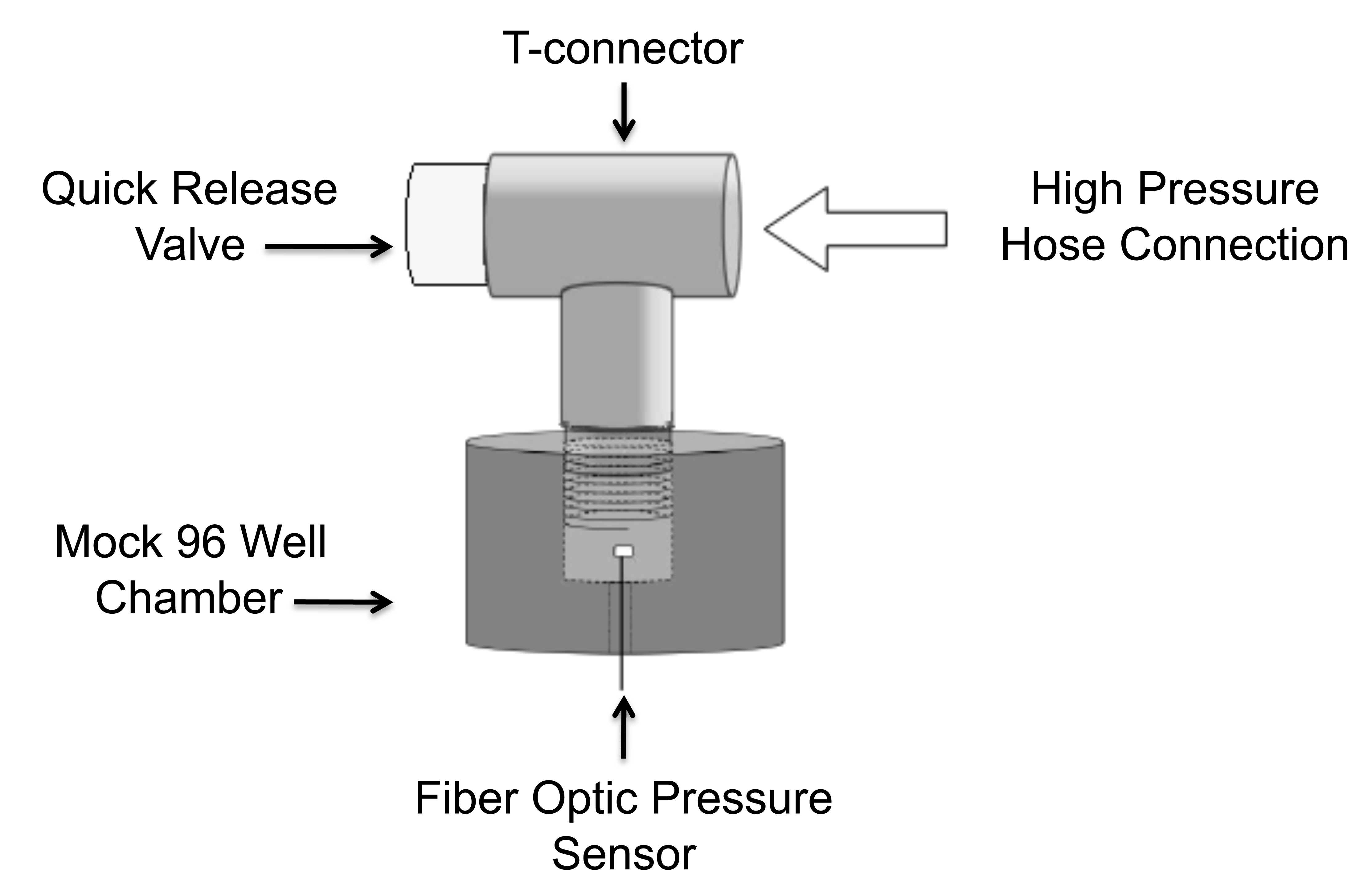

Supplement: Figure S1 — Schematic diagram of the T-connector installed onto the mock well chamber and fiber optic pressure sensor. (TIF) [file pone.0039421.s001.tif]

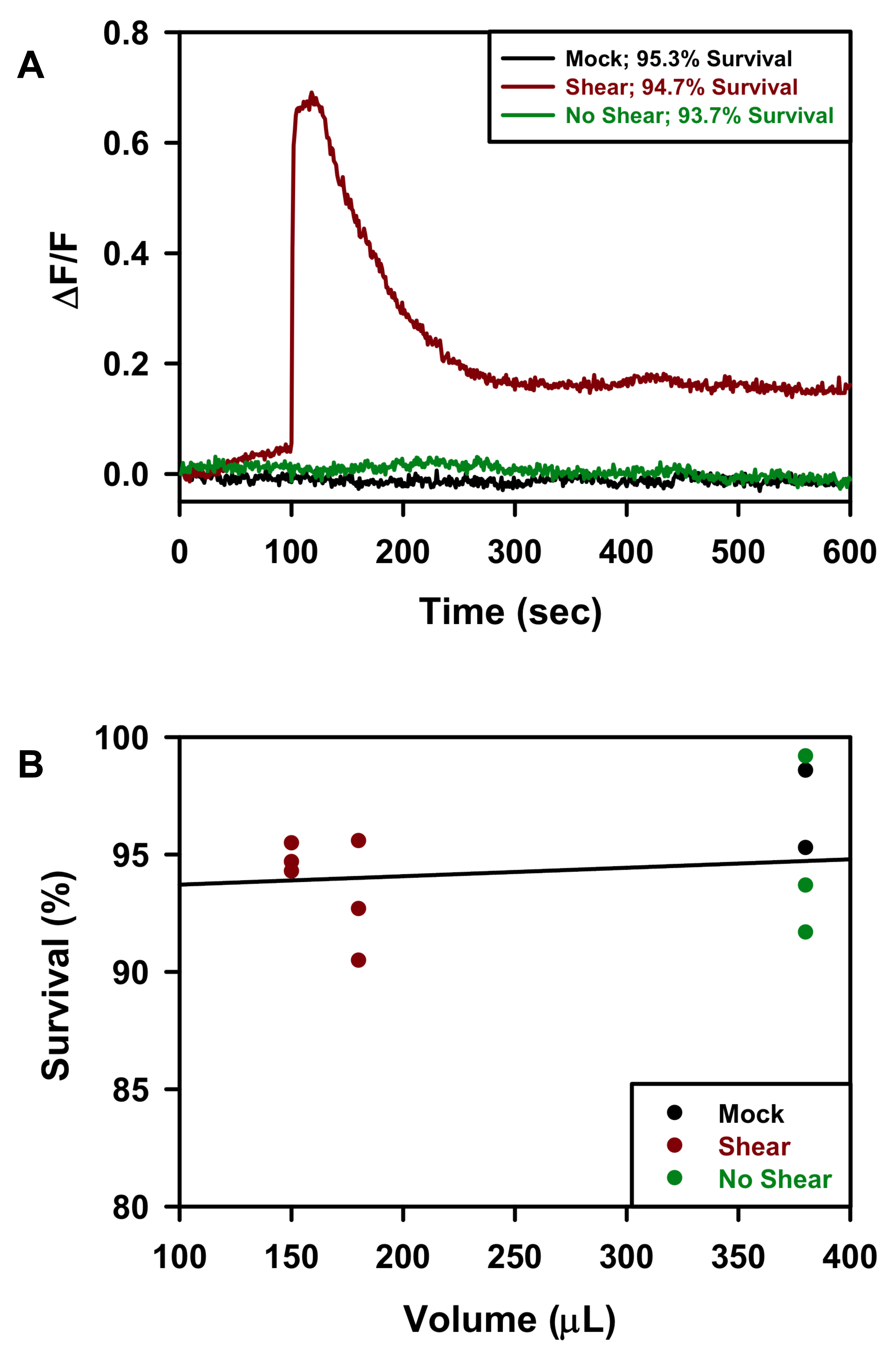

Supplement: Figure S2 — Cell survival is independent of blast conditions. A) ΔF/F for three examples of blast conditions, with and without shear and mock, no blast; survival at 20 hours was comparable for all three conditions, greater than ∼94%. B) The mean survival at 20 hours, evaluating 9,120 cells, was 94.7% +/−2.6% and ranged from 91.7%–99.2% with no correlation between survival and shear or blast (n = 11 experiments). (TIF) [file pone.0039421.s002.tif]

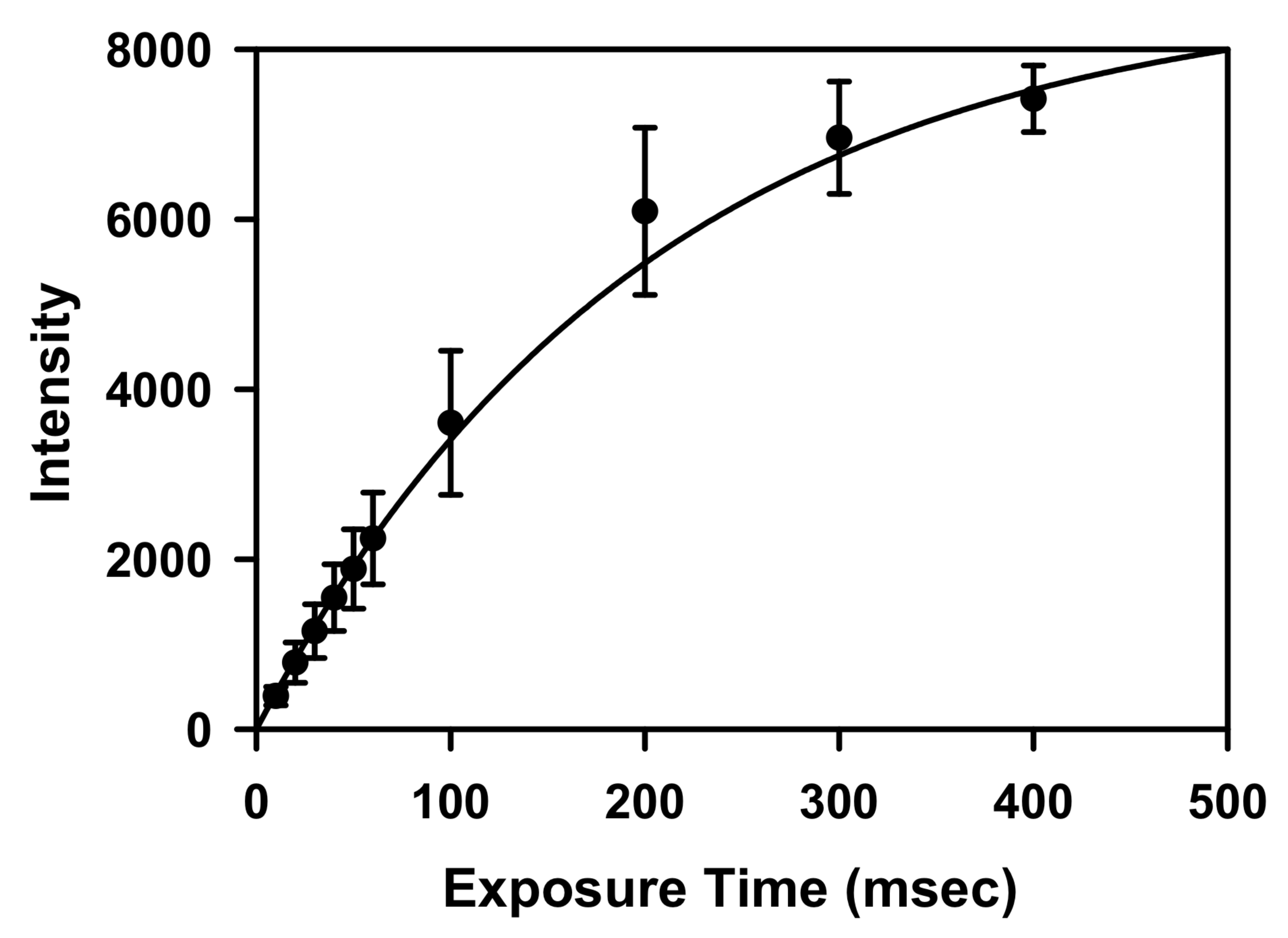

Supplement: Figure S3 — Stationary bead intensity as a function of exposure time. Error bars are standard deviations, and the solid line is the best fit to (Eq. (1)) with a = (8.73±0.25)×103 and b = (4.95±0.29)×10−3. (TIF) [file pone.0039421.s003.tif]

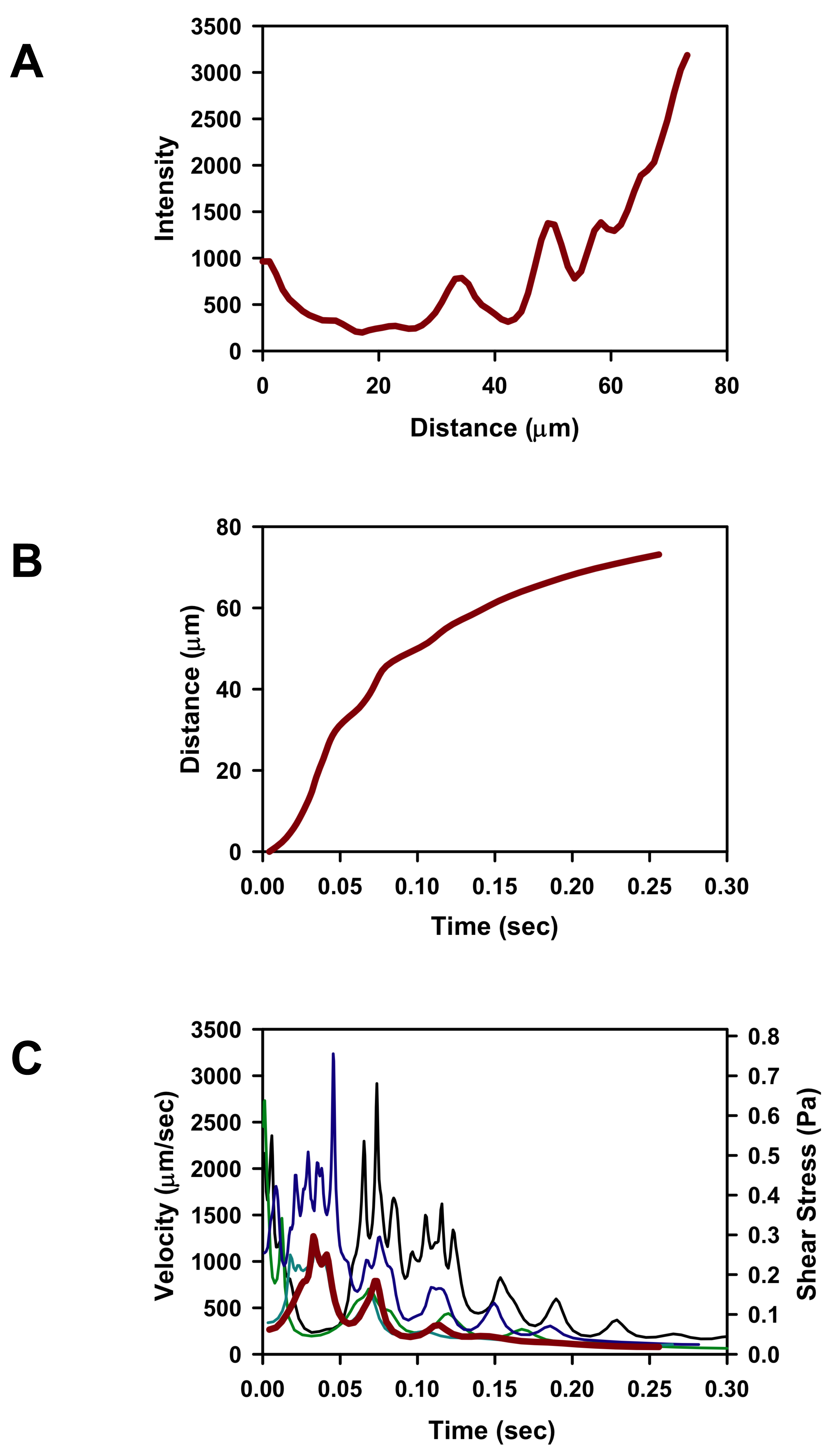

Supplement: Figure S4 — Derivation of shear stress profiles and representative examples. A) Intensity vs. trajectory length (Distance) for a moving bead captured in one image frame (see Figure 2). B) Time dependent trajectory length (Distance) vs. Time calculated using Eq. (2) and Eq. (3). C) Velocity and shear stress derived from the derivative of the time dependent trajectory length (thick red curve) and four other representative examples. (TIF) [file pone.0039421.s004.tif]
