# Supplementary material for: Shear Forces during Blast, Not Abrupt Changes in Pressure Alone, Generate Calcium Activity in Human Brain Cells
Source: PLoS One. 2012 Jun 29;7(6):e39421. doi: 10.1371/journal.pone.0039421 (PMC3387147; doi:10.1371/journal.pone.0039421)
Supplement: Text S1 — Supporting information text for “Shear Forces During Blast, Not Abrupt Changes in Pressure Alone, Generate Calcium Activity in Human Brain Cells” by Rea Ravin, Paul S. Blank, Alex Steinkamp, Shay Rappaport, Nitay Ravin, Ludmila Bezrukov, Hugo Guerrero-Cazares, Alfredo Quinones-Hinojosa, Sergey M. Bezrukov, and Joshua Zimmerberg. (DOC) [file pone.0039421.s005.doc]

Supporting Information Text for

Shear Forces During Blast, Not Abrupt Changes in Pressure Alone, Generate Calcium Activity in Human Brain Cells

Authors: Rea Ravin, Paul S. Blank, Alex Steinkamp, Shay Rappaport, Nitay Ravin, Ludmila Bezrukov, Hugo Guerrero-Cazares, Alfredo Quinones-Hinojosa, Sergey M. Bezrukov, and Joshua Zimmerberg

Measurement of Simulated Blast Parameters

To measure the simulated blast parameters, a mock 96 well chamber was constructed and threaded with the same tap used on the 96 well plate (Figure S1). Fiber optic pressure sensors (FISO Technologies FOP-MIV-BA-C1-F1-M2-R3-ST (0-150 PSI) or FOP-MEMs-1000PSI) were mounted in a hole in the bottom of the mock-chamber. The sensor was connected to a Veloce 50 controller (FISO Technologies) with a bandwidth of 200 kHz; pressure responses were displayed and recorded using a digital oscilloscope (Tektronix TDS 754D). This pneumatic device is capable of generating waveforms with high reproducibility, as demonstrated by the standard errors shown in Figure 1B.

Pressure Propagation is Independent of Well Volume

Fast transient pressure waves were generated with and without shear by varying the fluid volume within the 96 well chamber. Since the probe is easily damaged by shear stress and could not be used to measure the pressure with shear, it is important to consider whether the pressure changes at the bottom of the chamber, where the cells are attached, is the same for the different liquid volumes used. There are three reasons why the pressure properties are independent of the well volumes used: 1) for an ~ 1 kHz sound wave, the attenuation in water at 37 C is negligible for distances less than a cm 2) the pressure wave transmission depends upon the impedance mismatch between air and liquid and not on the length of the liquid volume and 3) the effective wavelength (i.e. the length scale where the pressure is homogeneous during the wave propagation) is $\lambda$ = v_s_$t$ (v_s_ is the sound velocity and $t$ the characteristic time). For our case, v_s_ > 1500 m/sec, and all time scales are longer than the rising time ($t$ ~ 0.1 msec). This leads to a characteristic wavelength longer than 15 cm that is much greater than the distances between the gas and well bottom used in both the shear and no shear conditions.

Estimating Shear Stress

Calibration

Frames of resting beads were taken at various exposure times between 10 ms and 400 ms. After Gaussian smoothing and “Rolling Ball” background subtraction the fluorescence intensity of each bead was measured from the center of mass (i.e., the pixels of highest intensity). As expected, it was found that the relations between the intensity ($I$) and exposure time () are linear for short time scales but begin to saturate at longer time scales. Figure S3 shows the averaged intensities versus exposure time for 24 beads. These values are fit to the asymptotic function

, Eq. (1)

with fitting parameters $a$ = (8.73 ± 0.25) x 10^3^ and $b$ =(4.95 ± 0.29) x 10^-3^; note, for short exposures,, the relation between intensity and time is linear.

Analyzing Bead Trajectories

After Gaussian smoothing and “Rolling Ball” background subtraction, the frame with the most significant displacements was selected, and the bead trajectories were analyzed (see Figure 2). Trajectories with overlaps were not evaluated. The basic data, illustrated in Figure S4 A, were obtained as intensity versus distance along the trajectory$S$ of one of the trajectories in Figure 2. Since a bead moves, each pixel accumulates signal from more than one point and therefore the intensity was rescaled as: $I\to(x/d)I$ where$x$ is the pixel size (≈1.4 µm) and d is the bead diameter (6 µm). The time a bead spent in one spot, was calculated by inverting the function in Eq. (1):

. Eq. (2)

The time it takes the bead to move to the n^th^ pixel was defined as:

, Eq. (3)

and the time-dependent trajectory length $S(t)$ was then numerically constructed (Figure S4 B). The velocity function was found as the first derivative of the trajectory length

. Eq. (4)

The shear stress in liquid medium above the surface is defined as

, Eq. (5)

where z is the height above the surface and$m$ is the medium viscosity. Assuming a linear growth of the velocity near the surface and the velocity at the surface equal to zero, we estimated the shear stress as

. Eq. (6)

The two characteristics, $v(t)$ and $t(t)$, of the shear flow obtained in one of the blasts with shear are plotted in Figure S4 C (thick red curve). Four additional examples of the velocity and shear stress are shown in Figure S4 C.
